# Ghrelin Mitigates High-Glucose-Induced Oxidative Damage and Apoptosis in Lens Epithelial Cells

**DOI:** 10.1155/2022/1373533

**Published:** 2022-12-21

**Authors:** Jie Bai, Ganggang Jiang, Mengdan Zhao, Shan Wang

**Affiliations:** ^1^Department of Ophthalmology, The Fourth Affiliated Hospital, Zhejiang University School of Medicine, Yiwu, 322000 Zhejiang, China; ^2^Department of Pharmacy, Women's Hospital, Zhejiang University School of Medicine, Hangzhou, 310006 Zhejiang, China; ^3^Department of Oral Pathology, School of Stomatology, Hainan Medical College, Haikou 571199, China

## Abstract

Oxidative stress induced by high glucose (HG) plays an important role in the mechanism of diabetic cataract. Evidence has shown that effects from oxidative stress induced damage of lens or human lens epithelial (HLE) cells. Antioxidant supplementation is a plausible strategy to avoid oxidative stress and maintain the function of lens. Ghrelin have been used in treatment of many diseases. In this study, we found that ghrelin attenuated HG-induced loss of cell viability, reduced oxidative damage, and cell apoptosis in HLE cells. Ghrelin inhibited apoptosis through the downregulation of Bax and the upregulation of Bcl-2. Our results suggest that ghrelin could be considered as a promising therapeutic intervention for diabetic cataract. We also observed rat lens transparent in cultured media and examined lens histopathological changes. The results showed that ghrelin could inhibit the histopathological injury of lenses and ultrastructural changes induced by HG. In conclusion, ghrelin may play a role in the treatment of ocular diseases involving diabetic cataract.

## 1. Introduction

Diabetic cataract is a complex disease of the lens that is related to high-glucose (HG) conditions [1]. Evidence has shown that oxidative stress induced by HG plays a significant role during the formation of diabetic cataract [2, 3]. Human lens epithelial (HLE) cells are monolayer subepithelial cells that are easily affected by the external environment [4, 5]. In this research, HG (30 mM) was used as an inducer of oxidative stress to explore the possible mechanism of antioxidative stress in HLE cells.

Antioxidant supplementation is a reasonable strategy to maintain the function of HLE cells and avoid oxidative stress [6]. However, to date, there is no effective way to inhibit oxidative stress-induced damage in HLE cells without adverse effects.

Ghrelin, a 28-amino-acid peptide hormone, is a recently discovered multifunctional gastrointestinal peptide hormone in rats and humans. Ghrelin confers many benefits to the human body: modulating tension stress, relieving anxiety, regulating glucose metabolism, stimulating appetite, enhancing gastric motility, improving cardiac function, and modulating immunity and inflammation [7–9]. It also has a variety of effects in the eye, including reducing intraocular pressure in an animal model of acute ocular hypertension, inhibiting the apoptosis of retinal ganglion cells (RGCs), protecting RGCs from rotenone invasion, and reducing the formation of retinal neovascularization [10–13]. Moreover, our previous studies showed that ghrelin considerably reduced H_2_O_2_-induced HLE cell apoptosis and impeded opacification of the lenses [14]. In this research, we investigated the role of ghrelin in protecting HLE cells from HG injury and explored the corresponding mechanisms.

## 2. Materials and Methods

### 2.1. Materials and Reagents

HLE cells obtained from the American Type Culture Collection (ATCC, Manassas, VA, USA), ghrelin (St Louis, MO, USA), 3-(4,5-dimethyl-thiazol-2-yl)-2,5-diphenyltetrazoliumbromide (MTT, Beyotime Institute of Biotechnology, Shanghai, China), Dulbecco's modified Eagle's medium (DMEM, Nanjing Jiancheng Bioengineering Institute, Nanjing, China), Annexin V−fluorescein isothiocyanate (FITC)/propidium iodide (PI) (BD Biosciences, Mountain View, CA, USA), trypan blue (Zeye Institute of Biotechnology, Shanghai, China), acridine orange/ethidium bromide (AO/EB, Solarbio of Biotechnology, Beijing, China), anti-Bax, anti-Bcl-2 antibodies (Santa Cruz Biotechnology, Santa Cruz, CA, USA), and hematoxylin–eosin (HE) kit (Jinqiao Biotechnology, Zhongshan, China).

### 2.2. Cell Viability Assay

HLE cells were cultured in DMEM media containing 10% heat-inactivated fetal bovine serum (FBS; HyClone, UT, USA), 100 U/mL streptomycin, and 100 U/mL penicillin for 24 h and then exposed to serum starvation for 12 h. To test the effect of ghrelin on cell viability, cells were cultured in normal medium (5.5 mM glucose), and different concentrations of ghrelin (0.01 *μ*M, 0.1 *μ*M, and 1 *μ*M) were added. To test the effect of ghrelin on HG-induced cell viability, ghrelin (0.1 *μ*M) was added 2 h before high-glucose medium (HG, 30 mM glucose) was added for 24 h. Then, the cells were incubated with 10 *μ*L of MTT (5 mg/mL) for 4 h, and absorption at 490 nm was measured by a microplate reader (SpectraMax iD3, CA, USA). Cell morphological changes were detected by light microscopy. In the following experiments, cells were divided into the control group, ghrelin group, HG group, and HG+ ghrelin group.

### 2.3. Basal ROS Level

Cells were stained with 2′,7′-dichlorofluorescin diacetate (DCF-DA) at 37°C for 30 min. Cells were then washed twice with prewarmed PBS. Fluorescence changes were measured by fluorescence spectrophotometry at 485 nm (excitation)/535 nm (emission).

### 2.4. Apoptosis Assays

The cells were then stained with trypan blue and acridine orange/ethidium bromide (AO/EB) dual staining and analyzed for the morphological examinations under the fluorescent microscope. Apoptotic rate (%) = number of apoptotic cells/total number of cells.

### 2.5. Annexin V-FITC/PI Fluorescent Staining

Cells were collected and centrifuged at 1,000 × g at 4°C for 5 min and then suspended in 400 *μ*L binding buffer (containing 5 *μ*L FITC and 5 *μ*L PI) for 20 min in the dark. The apoptosis percentage was detected by flow cytometry (Beckman Coulter, Inc., CA, USA), and the data were analyzed using a FACSCanto flow cytometer (Becton-Dickinson, Mountain View, CA, USA). The second quadrant and third quadrant of the flow cytometry data were calculated as the number of apoptotic cells. In addition, trypan blue staining was quantified as a second detection method for apoptotic cells. Trypan blue storage solution (0.4%) was prepared, and the dyeing time was controlled within 3 min.

### 2.6. Transmission Electron Microscopy

The cells were collected and fixed overnight in 2% glutaraldehyde solution at 4°C. Postfixation for 1 h with 1% OsO4, gradient alcohol dehydrated the pellets and then embedded in epoxy resin for 24 h. 70 nm thick sections were cut and collected with copper mesh. The slices were stained with uranyl acetate and lead citrate for 15 min and observed under transmission electron microscope (JEM 1200).

### 2.7. Western Blot Assay

The proteins were extracted and separated by SDS-PAGE and electrotransferred onto nitrocellulose membranes. Mouse anti-Bax polyclonal antibody (1 : 500, sc-7480) and mouse anti-Bcl-2 polyclonal antibody (1 : 500, sc-71022) were used as primary antibodies, and primary antibodies were detected using goat anti-mouse secondary antibodies (1 : 10000, Zhongshan Golden Bridge, Guangzhou, China). Blots were visualized using an enhanced chemiluminescence detection system, and the intensity of the protein bands was analyzed by Image Studio Lite software.

### 2.8. Lenses' Incubation and Observation of Lens Transparency

The animals in the present study were treated in accordance with the guidelines of the ARVO (Association for Research in Vision and Ophthalmology), and the animal experiments were approved by the Institutional Animal Care and Use Committee of Zhejiang University School of Medicine. 12 male Wistar rats (average weight 190 g) obtained from Zhejiang University (laboratory animal license number: SYXK (Zhejiang Province) 2018-0016) were anesthetized with diethyl ether and then killed by cervical dislocation. The eyes were enucleated, and the lenses were removed at once and immersed in 24-well plates containing 2 mL of DMEM medium in each well. A total of 24 lenses were divided into four groups (*n* = 6) as follows: group I: glucose 5.5 mM, group II: glucose 5.5 mM + ghrelin 1 *μ*M, group III: glucose 55 mM, and group IV: glucose 55 mM + ghrelin 1 *μ*M. Lenses were cultured at 37°C for 48 h, and the appearance of each lens was immediately photoed by placing the lens over a light source containing a grid, then removed from the culture medium and examined for morphological changes.

### 2.9. HE Staining

The paraffin-embedded lenses were dehydrated with a graded series of increasing ethanol concentrations, then were cut into 4 *μ*m thick sections and embedded in Epon mixture. HE staining was performed for morphological observation.

### 2.10. TUNEL Staining

After paraffin sections were dewaxed, TUNEL staining solution was added and incubated in 37°C for 2 h. The sections were put into 3% hydrogen peroxide solution prepared with methanol and incubated in the dark for 15 minutes; after the slices were dried, the freshly prepared DAB color developing solution was added, then control the developing time under the microscope; the positive is that the nucleus is brownish yellow, then wash the slices with tap water to stop the color developing.

### 2.11. Statistical Analysis

All experiments were performed in triplicate, and data was expressed as the mean ± standard deviation (SD). One-way analysis of variance (ANOVA) was used for statistical analysis with the aid of the software GraphPad Prism 6.0 (GraphPad Software, Inc., USA). A value of *P* < 0.05 was considered statistically significant.

## 3. Results

### 3.1. Ghrelin Inhibited HG-Induced HLE Cells' Cytotoxicity

**Figure 1:**
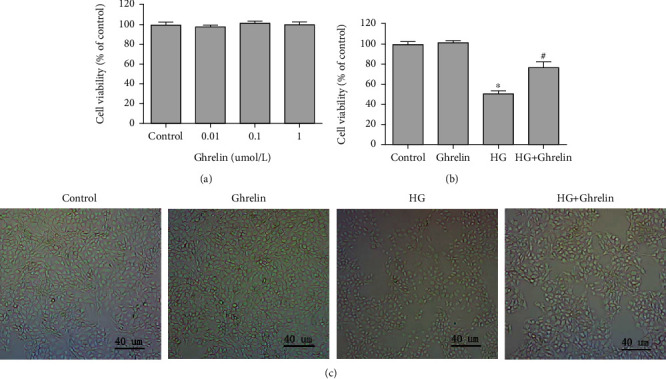
MTT assay for the survival rate of HLE cells. (a) HLE cells were treated with different concentrations of ghrelin (0 *μ*M, 0.01 *μ*M, 0.1 *μ*M, or 1 *μ*M) for 24 h. (b) Cells were pretreated with ghrelin (0.1 *μ*M) for 2 h and then exposed to HG for 24 h. (c) Morphological observation of HLE cells, about 400 cells were detected by light microscope in each picture. ^∗^*P* < 0.05 vs. control group; ^#^*P* < 0.05 vs. HG group.

Ghrelin concentrations of 0.01 *μ*M, 0.1 *μ*M, and 1 *μ*M were selected to detect the safety of ghrelin in HLE cells. The results showed that the concentration had no significant effect on cell viability (Figure 1(a)), the viability of cells was 95.53 ± 2.92%, 97.55 ± 2.04%, 101.4 ± 2.21%, and 99.75 ± 2.93%, respectively, and 0.1 *μΜ* (the highest value) ghrelin was chosen for subsequent experiments. HG destroyed cell viability, and exposure to 30 mM glucose resulted in an approximately 50% loss of cell viability (Figure 1(b)); ghrelin pretreatment of HLE cells showed protective effects against HG damage. The morphology of HLE cells was also examined. Cells in the control group and ghrelin group were uniformly spindle-shaped, but in the HG group, the space between cells decreased, and the cell shape decreased. In the HG + ghrelin group, changes in cell morphology were less pronounced (Figure 1(c)).

### 3.2. Ghrelin Decreased Intracellular Reactive Oxygen Species (ROS) Generation

**Figure 2:**
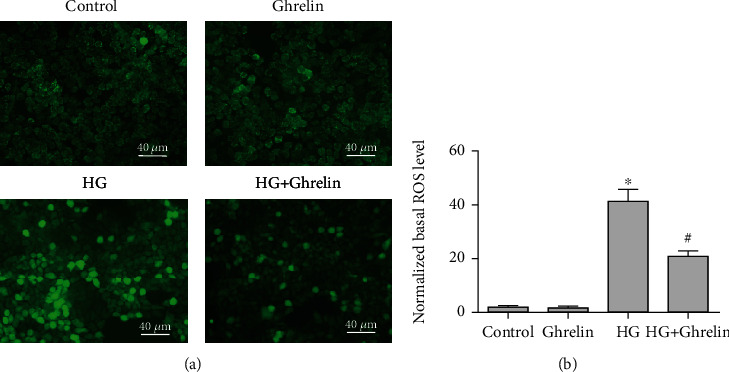
Ghrelin inhibited HG-induced HLE cell oxidative stress. (a) Effect of ghrelin on basal ROS level in HLE cells measured by H_2_DCFDA fluorescent probe. (b) Quantitative analyses of intracellular ROS generation in HLE cells. ^∗^*P* < 0.05 vs. control group; ^#^*P* < 0.05 vs. HG group.

To further examine the role of HG-induced oxidative stress in HLE cells, ROS levels within cells were detected using the H_2_DCFDA probe. The results showed that HG markedly enhanced the production of ROS; however, when ghrelin was added, there was a significant reduction in ROS levels (Figure 2).

### 3.3. Ghrelin Inhibited Cell Apoptosis

**Figure 3:**
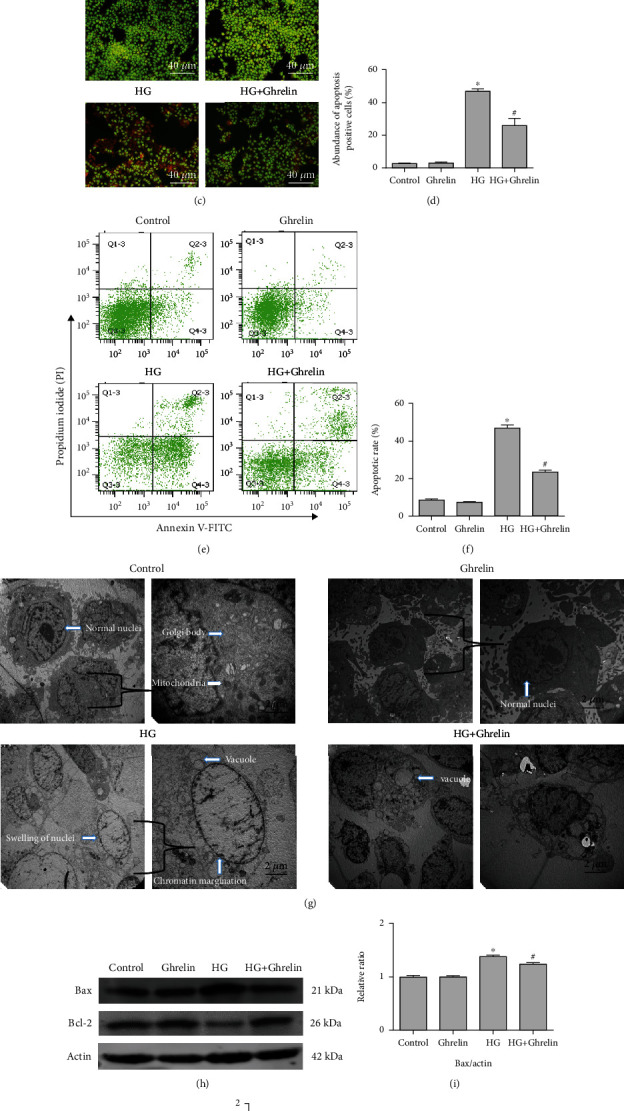
Ghrelin inhibited HG-induced HLE cell apoptosis. HLE cells were incubated with ghrelin (0.1 *μ*M) for 2 h and then exposed to HG for 24 h. (a) The number of trypan-positive cells was quantified, and at least 100 cells per dish were counted. (b) Quantitative analyses of the apoptosis rate in HLE cells. (c) AO/EB double stain of HLE cells after a treatment with ghrelin. (d) Quantitative analyses of the apoptosis rate in HLE cells. (e) Flow cytometric analysis was used to detect apoptosis rate. (f) Quantitative analyses of the apoptosis rate. (g) Ultrastructural changes in HLE cells induced by HG using transmission electron microscopy. (h) The expressions of Bax and Bcl-2 were detected by Western blot. (i) Quantitative analysis of Bax expression intensity relative to actin. (j) Quantitative analysis of Bcl-2 expression intensity relative to actin. ^∗^*P* < 0.05 vs. control group; ^#^*P* < 0.05 vs. HG group.

Trypan blue staining, AO/EB, and flow cytometry showed that ghrelin exerts its antiapoptosis effects. As shown in Figures 3(a)–3(f), HG-treated cells showed typical apoptotic morphological features such as blue nuclei by trypan blue staining and orange condensed nuclei by AO/EB staining. Similarly, the data of flow cytometry suggested that the percentage of apoptotic cells increased significantly after HG treatment, and ghrelin significantly decreased the cell apoptosis rate (HG, 47.10 ± 1.61%; HG + ghrelin, 23.53 ± 1.02%).

Ultrastructural changes in HLE cells were detected by transmission electron microscopy. In control group, the ultrastructure was normal, the nuclei were intact, and the morphology of mitochondria and Golgi bodies was normal. In contrast, cells treated with HG were swollen, and vacuoles were found; swellings of nucleus were obvious; organelles were ruptured or fragmented. The images indicated that HG-treated cells were undergoing an apoptotic procedure. The ultrastructure of cells in HG + Ghrelin group was significantly improved than that in HG group (Figure 3(g)). Transmission electron microscopy showed that ghrelin could preserve the ultrastructural changes induced by HG.

Western blot analysis showed that ghrelin pretreatment decreased the protein expression of proapoptotic Bax and enhanced the expression of antiapoptotic Bcl-2 (Figures 3(h) and 3(i)).

### 3.4. Visual Examination and Histological Analysis

**Figure 4:**
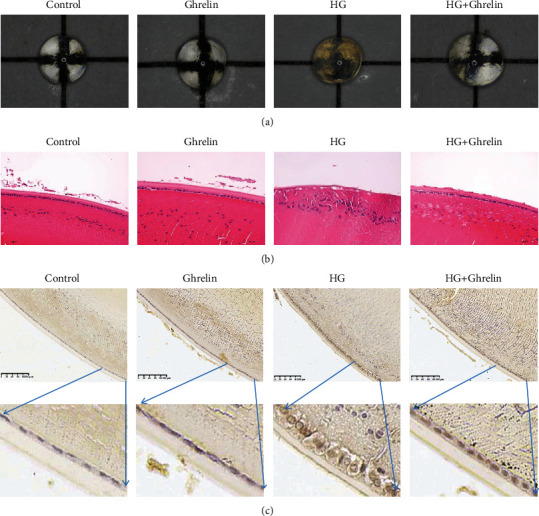
Representative illustrations of anticataract activity of ghrlein. (a) Lenses' photos were taken over a grid. (b) Changes in the lenses' structure stained with hematoxylin and eosin (×400). (c) Changes in the lenses' structure stained with TUNEL (×400).

Appearance of lenses was captured by microscope. It can be clearly seen that lenses in control group and ghrelin group remained transparent during the incubation period, and the gridlines under the lenses were clearly visible. Lenses in HG group showed extensive thick pacification involving the entire lens, with total clouding of gridlines, which nearly cannot be seen, and the increased lens opacity induced by diabetic condition was attenuated by treatment with ghrelin; lenses in HG + ghrelin group showed minimal clouding of gridlines, and gridlines are still visible (Figure 4(a)). HE staining showed an (closely and regularly) orderly arrangement of fiber cells in the control group and ghrelin group, HG treatment induced the irregular arrangement of fiber cells, and the lenses exhibited expanded extracellular lacunae, and empty cells can be seen between cells (Figure 4(b)). TUNEL staining showed that the nuclei in control group and ghrelin group were blue and uniform in size; nuclei in HG group are large and deeply stained brown, and there were more vacuoles between lens cells; in HG + ghrelin group, the number of brown cells decreased (Figure 4(c)), which suggested that the morphological changes induced by HG were blocked by ghrelin.

## 4. Discussion

Diabetic cataract, also known as metabolic cataract, often occurs in both eyes and progresses rapidly; the lens may exhibit complete opacity in days, weeks, or months [15]. In diabetes mellitus, blood sugar is increased, and glucose in the lenses is also increased. Glucose is converted into sorbitol, which cannot pass through the lenses' capsule membrane and accumulates in the lenses in large quantities. Osmotic pressure in the lenses then increases, HLE cells swell and denature, and the lenses become opaque [16, 17].

HG status is associated with metabolic disorders such as type 2 diabetes mellitus. The HG condition induces oxidative stress, resulting in cell damage [18]. Many investigations have indicated that ROS generated by HG leads to protein degradation, which is similar to that observed with diabetic cataract [19, 20].

The most effective way to delay the progression of cataracts is to supplement antioxidants. Drugs that inhibit the level of ROS in HLE cells are considered to be effective; they are the primary method to treat oxidative damage to the lenses [21]. Ghrelin plays an important role in cardiovascular, nervous system, immune, metabolic, reproductive, endocrine, and other physiological processes [22–25]. Ghrelin is an endogenous “brain-gut” peptide and is safe for humans. It could reduce apoptosis induced by various pathological stimuli, such as hydrogen peroxide and high glucose [14, 26, 27]. In this study, ghrelin significantly inhibited HG-induced cell oxidative stress and injury.

The antiapoptosis effect of ghrelin in the eye has been reported by many researchers. Shenwen Liu showed that ghrelin can protect rat retinal ganglion cells against rotenone via restoring mitochondrial functions and inhibiting apoptosis in RGC-5 cells [28]. Shimada tested the effect of Des-ghrelin in human retinal microvascular endothelial cells and found that Des-ghrelin could reduce hydrogen peroxide-induced damage by decreasing ROS production, increasing antioxidant enzymes such as MnSOD and CAT expression [29]. Our previous results also confirmed the protective effect of ghrelin on lenses tissue and HLE cells [14]. In this experiment, we changed the inducer of oxidative damage from H_2_O_2_ to HG and used different experimental methods to further verify the protective effect of ghrelin on HLE cells.

We further detected the ultrastructural changes in HLE cells by transmission electron microscopy. In control group, Golgi body is arched or hemispherical in appearance and composed of many flat vesicles, and mitochondria exist in the cytoplasm of cells and are generally in the shape of short rods or globe. Cells in HG group showed morphological features of apoptosis, including formation of vacuoles and swelling of nucleus. Treatment with ghrelin preserved the normal cell morphology.

Bax and Bcl-2 can regulate apoptosis, and they are two important members of Bcl-2 family. The decrease of Bax and increase of Bcl-2 level indicate that the resistance of cells to apoptosis is enhanced, which should be the mark of protective drugs. The results showed that ghrelin inhibited apoptosis by downregulating Bax and upregulating Bcl-2 in HG-treated HLE cells. In addition, pretreatment with ghrelin decreased the expression of Bax and enhanced the expression of Bcl-2 protein, which suggest that ghrelin may rearrange Bax/Bcl-2 ratio by inactivate the intrinsic signaling pathway.

Rat lenses' organ culture studies have been a powerful experimental tool to elucidate the effect of statins on cataract formation. In vitro lenses' organ culture showed that HG can promote lenses turbidity, and the clarity of cataractous rat lenses was improved after ghrelin intervention; histological examination further verified that HG can damage lens epithelial cells, and lens epithelial cells disorder were significantly improved when ghrelin added. These results suggest that ghrelin cannot only delay the occurrence of HG-induced oxidative damage in diabetic cataract but also protect HLE cells and reduce cell apoptosis.

In general, we concluded that HG could induce HLE cell apoptosis; pretreatment with ghrelin induced a substantial protection against HG-induced apoptosis, and this protection likely occurs through a reduction in Bax/Bcl-2 ratio. These multifunctional effects and safety of ghrelin make it a potential new therapy for patients with diabetic cataract.

## Data Availability

The datasets used and/or analyzed during the current study are available from the corresponding authors on reasonable request.

## Additional Points

*Key Messages*. (1) Ghrelin inhibits apoptosis by downregulating Bax and upregulating Bcl-2. (2) Ghrelin could be a promising treatment for diabetic cataract.

## References

[1] Higashi Y., Higashi K., Mori A., Sakamoto K., Ishii K., Nakahara T. (2018). Anti-cataract effect of resveratrol in high-glucose-treated streptozotocin-induced diabetic rats. *Biological & Pharmaceutical Bulletin*.

[2] Li D., Liu G. Q., Lu P. R. (2019). High glucose: activating autophagy and affecting the biological behavior of human lens epithelial cells. *International Journal of Ophthalmology*.

[3] Chen M. F., Liou S. S., Hong T. Y., Kao S. T., Liu I. M. (2019). Gigantol has protective effects against high glucose-evoked nephrotoxicity in mouse glomerulus mesangial cells by suppressing ROS/MAPK/NF-*κ*B signaling pathways. *Molecules*.

[4] Chen L. H., Chen Y. Z., Ding W. (2022). Oxidative stress-induced TRPV2 expression increase is involved in diabetic cataracts and apoptosis of lens epithelial cells in a high-glucose environment. *Cells*.

[5] Li X., Meng F., Li H., Hua X., Wu L., Yuan X. (2019). L-carnitine alleviates oxidative stress-related damage via MAPK signaling in human lens epithelial cells exposed to H_2_O_2_. *International Journal of Molecular Medicine*.

[6] Bai J., Yang Y., Wu D., Yang F. (2021). SS-31 protect retinal pigment epithelial cells from H_2_O_2_-induced cell injury by reducing apoptosis. *Clinical and Experimental Pharmacology & Physiology*.

[7] Jeon S. G., Hong S. B., Nam Y. (2019). Ghrelin in Alzheimer's disease: pathologic roles and therapeutic implications. *Ageing Research Reviews*.

[8] Dos-Santos R. C., Reis L. C., Perello M., Ferguson A. V., Mecawi A. S. (2019). The actions of ghrelin in the paraventricular nucleus: energy balance and neuroendocrine implications. *Annals of the New York Academy of Sciences*.

[9] Buntwal L., Sassi M., Morgan A. H., Andrews Z. B., Davies J. S. (2019). Ghrelin-mediated hippocampal neurogenesis: implications for health and disease. *Trends in Endocrinology and Metabolism*.

[10] Rocha-Sousa A., Pereira-Silva P., Tavares-Silva M. (2014). Identification of the ghrelin-GHSR 1 system and its influence in the modulation of induced ocular hypertension in rabbit and rat eyes. *Peptides*.

[11] Zaniolo K., Sapieha P., Shao Z. (2011). Ghrelin modulates physiologic and pathologic retinal angiogenesis through GHSR-1a. *Invest Ophthalmol Vis Sci*.

[12] Zhu K., Zhang M. L., Liu S. T. (2017). Ghrelin attenuates retinal neuronal autophagy and apoptosis in an experimental rat glaucoma model. *Investigative Ophthalmology & Visual Science*.

[13] Liu Y., Xing Y. X., Gao X. Y., Kuang H. Y., Zhang J., Liu R. (2018). Obestatin prevents H_2_O_2_-induced damage through activation of TrkB in RGC-5 cells. *Biomedicine & Pharmacotherapy*.

[14] Bai J., Yang F., Dong L., Zheng Y. (2017). Ghrelin protects human lens epithelial cells against oxidative stress-induced damage. *Oxidative Medicine and Cellular Longevity*.

[15] Thiagarajan R., Varsha M. K. N. S., Srinivasan V., Ravichandran R., Saraboji K. (2019). Vitamin K1 prevents diabetic cataract by inhibiting lens aldose reductase 2 (ALR2) activity. *Scientific Reports*.

[16] Yang J., Zhao S., Tian F. (2020). SP1-mediated lncRNA PVT1 modulates the proliferation and apoptosis of lens epithelial cells in diabetic cataract via miR-214-3p/MMP2 axis. *Journal of Cellular and Molecular Medicine*.

[17] Han Z. H., Wang F., Wang F. L., Liu Q., Zhou J. (2018). Regulation of transforming growth factor *β*-mediated epithelial-mesenchymal transition of lens epithelial cells by c-Src kinase under high glucose conditions. *Experimental and Therapeutic Medicine*.

[18] Luc K., Schramm-Luc A., Guzik T. J., Mikolajczyk T. P. (2019). Oxidative stress and inflammatory markers in prediabetes and diabetes. *J. Physiology Pharmacology*.

[19] Burgos-Morón E., Abad-Jiménez Z., Marañón A. M. (2019). Relationship between oxidative stress, ER stress, and inflammation in type 2 diabetes: the battle continues. *Journal of Clinical Medicine*.

[20] Newsholme P., Cruzat V. F., Keane K. N., Carlessi R., de Bittencourt P. I. (2016). Molecular mechanisms of ROS production and oxidative stress in diabetes. *The Biochemical Journal*.

[21] Chen Y. Y., Wu T. T., Ho C. Y. (2019). Dapagliflozin prevents NOX- and SGLT2-dependent oxidative stress in lens cells exposed to fructose-induced diabetes mellitus. *International Journal of Molecular Sciences*.

[22] Mani B. K., Zigman J. M. (2017). Ghrelin as a survival hormone. *Ghrelin as a Survival Hormone*.

[23] Fang C., Xu H., Guo S., Mertens-Talcott S. U., Sun Y. (2018). Ghrelin signaling in immunometabolism and inflamm-aging. *Advances in Experimental Medicine and Biology*.

[24] Tokudome T., Otani K., Miyazato M., Kangawa K. (2019). Ghrelin and the heart. *Peptides*.

[25] Mani B. K., Shankar K., Zigman J. M. (2019). Ghrelin's relationship to blood glucose. *Endocrinology*.

[26] El Zein N., Abdallah M. S., Daher C. F. (2019). Ghrelin modulates intracellular signalling pathways that are critical for podocyte survival. *Cell Biochemistry and Function*.

[27] Liao P., Yang D., Liu D., Zheng Y. (2018). GLP-1 and ghrelin attenuate high glucose/high lipid-induced apoptosis and aenescence of human microvascular endothelial cells. *Cellular Physiology and Biochemistry*.

[28] Liu S., Chen S., Ren J., Li B., Qin B. (2018). Ghrelin protects retinal ganglion cells against rotenone via inhibiting apoptosis, restoring mitochondrial function, and activating AKT-mTOR signaling. *Europe*.

[29] Shimada T., Furuta H., Doi A. (2014). Des-acyl ghrelin protects microvascular endothelial cells from oxidative stress-induced apoptosis through sirtuin 1 signaling pathway. *Metabolism*.

